# Associations of Alzheimer’s disease risk variants with gene expression, amyloidosis, tauopathy, and neurodegeneration

**DOI:** 10.1186/s13195-020-00755-7

**Published:** 2021-01-08

**Authors:** Meng-Shan Tan, Yu-Xiang Yang, Wei Xu, Hui-Fu Wang, Lin Tan, Chuan-Tao Zuo, Qiang Dong, Lan Tan, John Suckling, Jin-Tai Yu, Alzheimer’s Disease Neuroimaging Initiative

**Affiliations:** 1 Department of Psychiatry, University of Cambridge, Cambridge, UK; 2 Department of Neurology, Qingdao Municipal Hospital, Qingdao University, Qingdao, China; 3 Department of Neurology and Institute of Neurology, Huashan Hospital, Shanghai Medical College, Fudan University, 12th Wulumuqi Zhong Road, Shanghai, 200040 China; 4 PET Center, Huashan Hospital, Fudan University, Shanghai, China

**Keywords:** Alzheimer disease, Amyloid, Tau, Neurodegeneration, Risk variants, Gene expression

## Abstract

**Background:**

Genome-wide association studies have identified more than 30 Alzheimer’s disease (AD) risk genes, although the detailed mechanism through which all these genes are associated with AD pathogenesis remains unknown. We comprehensively evaluate the roles of the variants in top 30 non-*APOE* AD risk genes, based on whether these variants were associated with altered mRNA transcript levels, as well as brain amyloidosis, tauopathy, and neurodegeneration.

**Methods:**

Human brain gene expression data were obtained from the UK Brain Expression Consortium (UKBEC), while other data used in our study were obtained from the Alzheimer’s Disease Neuroimaging Initiative (ADNI) cohort. We examined the association of AD risk allele carrier status with the levels of gene expression in blood and brain regions and tested the association with brain amyloidosis, tauopathy, and neurodegeneration at baseline, using a multivariable linear regression model. Next, we analyzed the longitudinal effects of these variants on the change rates of pathology using a mixed effect model.

**Results:**

Altogether, 27 variants were detected to be associated with the altered expression of 21 nearby genes in blood and brain regions. Eleven variants (especially novel variants in *ADAM10*, *IGHV1-68*, and *SLC24A4/RIN3*) were associated with brain amyloidosis, 7 variants (especially in *INPP5D*, *PTK2B*) with brain tauopathy, and 8 variants (especially in *ECHDC3*, *HS3ST1*) with brain neurodegeneration. Variants in *ADAMTS1*, *BZRAP1-AS1*, *CELF1*, *CD2AP*, and *SLC24A4/RIN3* participated in more than one cerebral pathological process.

**Conclusions:**

Genetic variants might play functional roles and suggest potential mechanisms in AD pathogenesis, which opens doors to uncover novel targets for AD treatment.

**Supplementary Information:**

The online version contains supplementary material available at 10.1186/s13195-020-00755-7.

## Background

Alzheimer’s disease (AD) is highly heritable, with late-onset AD (LOAD) showing heritability of 58–79% [1]. Previous large-scale genome-wide association studies (GWASs) have discovered more than 20 AD gene variants that confer risk for LOAD [2–7]. *APOE* is still the strongest genetic risk factor for LOAD, responsible for a 3- to 15-fold increase in risk [8]. Yet these confirmed variants only account for a small portion of disease heritability. In the search for additional LOAD risk variants, recent GWAS meta-analyses [9–11] identified totally more than 30 AD risk genes. The identification of these novel gene variants might provide valuable insights into the molecular mechanisms with important roles in AD pathogenesis.

Although most of the non-*APOE* AD-associated genetic variants described to date are located in intronic or noncoding regions, these variants still could affect the nearby gene expression and exert protective or disease-inducing effects [12, 13]. In addition, AD develops following a long pre-clinical phase with abnormal neuropathological biomarkers [14]. Recently, the National Institute on Aging and Alzheimer’s Association (NIA-AA) published an updated research framework [15] that provides a flexible platform to generate or test hypotheses concerning different pathologic processes of AD, which defines AD by neuropathological biomarkers in three categories: amyloidosis [A] biomarkers, particularly cortical amyloid-PET ligand binding or low CSF Aβ_42_; tauopathy [T] biomarkers, particularly elevated CSF phosphorylated tau (pTau) or cortical tau-PET ligand binding; and neurodegeneration [N] biomarkers, particularly elevated CSF total tau (tTau), diminished ^18^F-fluorodeoxyglucose (FDG)-PET, or brain structural atrophy on MRI.

Many of the early identified AD variants have been associated with expression levels of their nearby genes and implicated in brain amyloidosis and neurodegeneration [12, 16, 17]. Relatively, fewer studies have reported associations of these top AD risk gene variants with brain tauopathy [18–20], and most of these studies focused on a single variant, or a few variants. More importantly, the precise disease-associated mechanisms of the novel genetic variants identified in the recent GWAS meta-analysis remain unknown. Here, we report a comprehensive analysis of the associations of the variants in the top 30 non-*APOE* AD risk genes from current large-scale GWAS studies of the transcript expression levels, and the pathological processes of brain amyloidosis, tauopathy, and neurodegeneration, using the baseline and follow-up data from AD-related CSF, PET, and MRI measures. Understanding the mechanisms by which these variants contribute to AD risk will lead to a better understanding of the disease-associated mechanisms and help uncover novel therapeutic avenues.

## Methods

### Study design and participants

Human brain gene expression data were obtained from Braineac dataset, UK Brain Expression Consortium (UKBEC), and included 10 brain regions from 134 neuropathologically normal individuals of European descent. The 10 brain regions were the cerebellar cortex (CRBL), frontal cortex (FCTX), hippocampus (HIPP), medulla (MEDU), occipital cortex (OCTX), putamen (PUTM), substantia nigra (SNIG), temporal cortex (TCTX), thalamus (THAL), and intralobular white matter (WHMT). In the Braineac dataset, Affymetrix GeneChip Human exon 1.0ST arrays were used to measure the gene expression in transcript levels. Detailed information on these methods is described in the Braineac database [21]. Genomic DNA for individuals from UKBEC was extracted from sub-dissected samples (100–200 mg) of human post-mortem brain tissue using Gentra Puregene Kit (Qiagen, UK). Samples from every individual were run on the genotype chip (the Illumina Infinium Omni1-Quad BeadChip). The BeadChips were scanned using an iScan (Illumina, USA) with an AutoLoader (Illumina, USA). GenomeStudio v.1.8.X (Illumina, USA) was used for analyzing the data and generating SNP calls. All other data used in this study were obtained from Alzheimer’s Disease Neuroimaging Initiative (ADNI) database (http://adni.loni.usc.edu). ADNI was launched in 2003 as a public-private partnership, led by Principal Investigator Michael W. Weiner, MD, VA Medical Center and University of California-San Francisco. Our analyses included all individuals with diagnosed AD, mild cognitive impairment (MCI), and normal cognition (NC), with clinical information, GWAS data, and gene expression data from peripheral blood (Affymetrix Human Genome U219 Array platform), or longitudinal AD-related CSF, PET, and MRI data. Furthermore, we selected only non-Hispanic white individuals to avoid population stratification effects which can lead to spurious findings. In total, 1183 individuals at baseline were included in our study (Table 1). Among them, 739 individuals had gene expression data (Additional file 1: Figure S1).

**Table 1:** Demographic characteristics of study subjects in ADNI

Characteristics	NC (*N* = 339)	MCI (*N* = 639)	AD (*N* = 205)
Age (mean years ± SD)	75.15 ± 5.35	73.44 ± 7.52	75.53 ± 7.81
Gender (*n* (%))			–
Male	177 (52.21)	389 (60.88)	117 (57.07)
Female	162 (47.79)	250 (39.12)	88 (42.93)
Education (mean years ± SD)	16.30 ± 2.68	15.89 ± 2.84	15.03 ± 2.96
*APOE* status (*n* (%))			
*APOE ε2/ε2*,*ε2/ε3*,*ε3/ε3*	247 (72.86)	325 (50.86)	66 (32.20)
*APOE ε2/ε4*,*ε3/ε4*	83 (24.48)	250 (39.12)	101 (49.27)
*APOE ε4/ε4*	9 (2.65)	64 (10.02)	38 (18.54)
MMSE (means ± SD)	29.07 ± 1.12	27.60 ± 1.78	23.29 ± 2.03

*Abbreviations*: *AD* Alzheimer’s disease, *ADNI* Alzheimer’s Disease Neuroimaging Initiative, *MCI* mild cognitive impairment, *MMSE* Mini-Mental State Exam scores, *N* number, *NC* normal cognition, *SD* standard deviation

### Gene variant selection and imputation

The ADNI-1, ADNI-2, and ADNI–Grand Opportunity (GO) participants were genotyped according to the manufacturer’s protocol. We focused on well-established AD risk genes identified in AD GWASs available to date (Additional file 1: Table S1), which yielded a total of 68 variants. Missing genotypes were imputed using the Beagle software with the HapMap GRCh37 as a reference. Among them, 19 of our genes were represented by more than one variant. We performed linkage disequilibrium (LD) analyses followed by Cohen kappa (*κ*) statistics. When choosing between 2 variants with significant overlap (high *r*^2^ and high *κ*), we retained the variant with the smallest amount of missing data. Our final number of non-*APOE* AD variants was thus reduced to 42 (Additional file 1: Table S2). More detailed information is described in Additional file 1 .

### CSF measurements

CSF Aβ_42_ and pTau were measured at the ADNI Biomarker Core Laboratory (University of Pennsylvania) using the multiplex xMAP Luminex platform (Luminex Corp, Austin, TX) with Innogenetics (INNO-BIA AlzBio3; Ghent, Belgium; for research use only reagents) immunoassay kit-based reagents. All CSF biomarker assays were performed in duplicate and averaged.

### AV45-PET/AV1415-PET data acquisition and analyses

A detailed description of PET image acquisition and processing can be found at http://adni.loni.usc.edu/datasamples/pet/. The AV45-PET (amyloid-PET) and AV1415-PET (tau-PET) standardized uptake value ratios (SUVRs) were formed by normalizing composite multi-region target regions of interest (ROIs) to the cerebellar crus gray matter. The amyloid-PET target meta-ROI included the frontal, anterior cingulate, precuneus, and parietal cortex [22]. The tau-PET target meta-ROI used in the primary analysis included the amygdala, entorhinal cortex, fusiform, parahippocampal, and inferior temporal and middle temporal gyri [23].

### FDG-PET data acquisition and analyses

The cerebral metabolic rate for glucose (CMRgl) data on FDG-PET was downloaded from the ADNI dataset. Mean FDG uptake was averaged from 5 meta-ROIs including the right and left angular gyri, right and left inferior temporal regions, and bilateral posterior cingulate. PET images were spatially normalized in Statistical Parametric Mapping (SPM) to the MNI PET template. We intensity-normalized each meta-ROI mean by dividing it by the pons/vermis reference region mean.

### Structural MRI data

Hippocampal volume (HV) and estimated intracranial volume (eICV) were performed from T1-weighted MRI acquired with a Siemens Trio 3.0 T or 1.5 T scanner. Regional volume estimates were processed using the Freesurfer software (https://surfer.nmr.mgh.harvard.edu). HV was adjusted for eICV using the following equation: Adjusted HV (HVa) = Raw HV – *b* (eICV – Mean eICV), where *b* is the regression coefficient when HV is regressed against eICV.

### Statistical analyses

Clinical and demographic characteristics for each variant were compared using *t* tests or *χ*^2^ tests with 2-sided *P* values, as appropriate. First, a multivariable linear regression model was used to analyze the association of AD risk allele carrier status with the gene expression levels, using the genes that have been annotated to those variants by GWAS. Age and gender as covariates were included in the model and tested for statistical significance. A multivariable linear regression model was also used to analyze the association of AD risk allele carrier status with brain amyloidosis, tauopathy, and neurodegeneration, based on AD-related CSF, PET, and MRI data at baseline. Age, gender, educational level, *APOEε4* genotype, and diagnosis were included as covariates. Then, we analyzed the longitudinal effects of these variants on the change rates of pathologically characteristic data above, using a multivariable linear mixed effect model with fixed effects of time (year) from baseline, AD risk allele carrier status, and interaction between time from baseline and AD risk allele carrier status. The model included random slope and intercept terms for each participant with age, gender, educational level, *APOEε4* genotype, and diagnosis as covariates. All statistical analyses were conducted using R statistical software. We defined associations with false discovery rate (FDR)-adjusted *P* values < 0.05 as statistically significant using the Benjamini-Hochberg procedure.

## Results

### Association of variants with gene expression in the blood

**Fig. 1:**
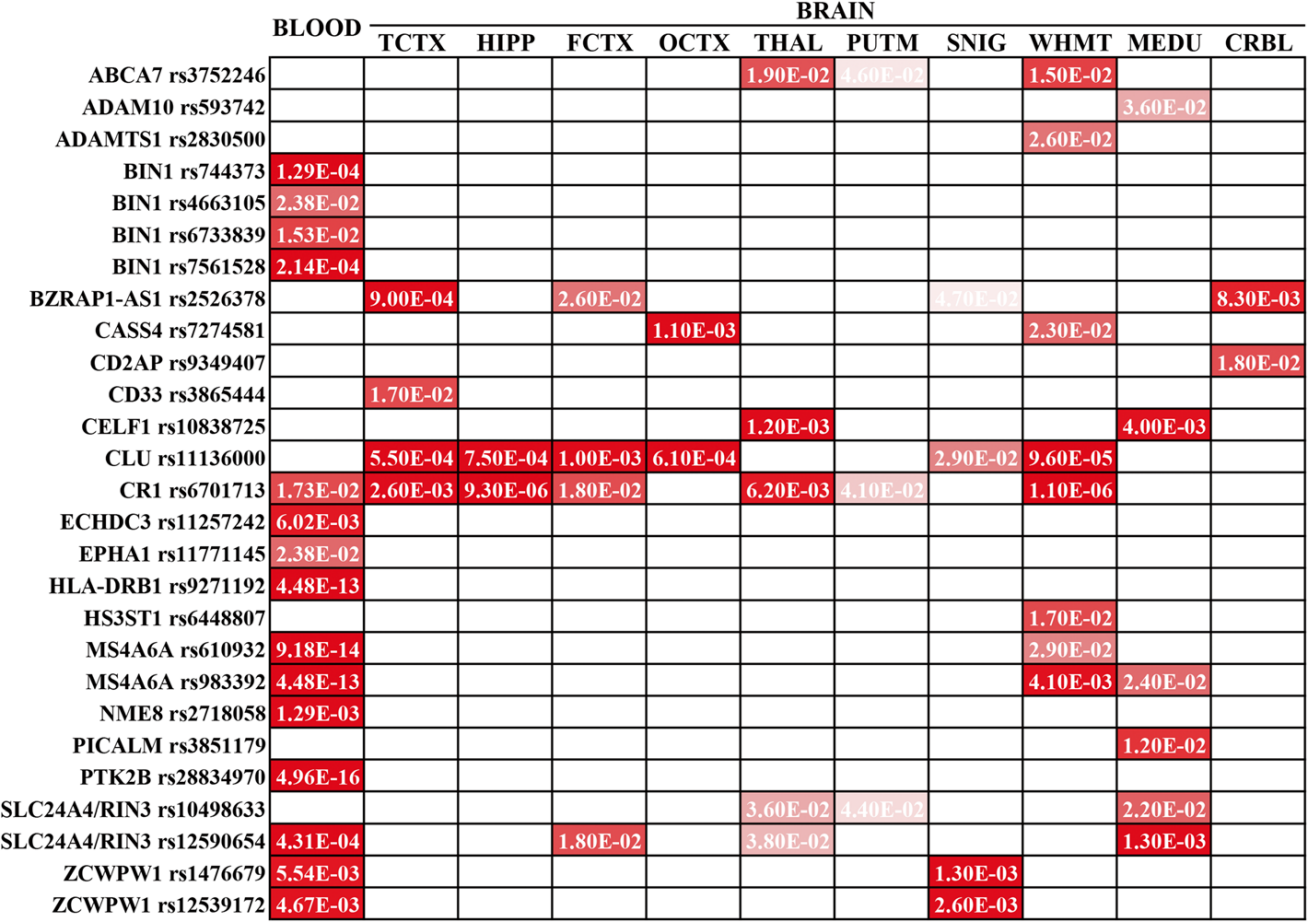
Association of the variants with gene expression in peripheral blood and brain regions. The significant associations between the variants and the levels of gene expression in blood were identified from the ADNI database, and the significant associations in specific brain regions were obtained from the Braineac dataset. FDR-adjusted *P* values with statistical significance are shown. Abbreviations: CRBL, cerebellar cortex; FCTX, frontal cortex; HIPP, hippocampus; MEDU, medulla; OCTX, occipital cortex; PUTM, putamen; SNIG, substantia nigra; TCTX, temporal cortex; THAL, thalamus; WHMT, intralobular white matter

The associations between AD risk allele carrier status and gene expression in the blood are shown in Additional file 1: Table S3. Figure 1 shows those associated variants that reached FDR-adjusted significance level. In total, 15 variants were detected to be associated with the altered expression in blood of 10 nearby genes. The minor alleles of 5 variants in *CR1*, *ECHDC3*, *MS4A6A*, and *NME8* were associated with decreased expressions, while the other 10 variants in *BIN1*, *EPHA1*, *HLA-DRB1*, *PTK2B*, *SLC24A4/RIN3*, and *ZCWPW1* associated with increased expressions.

### Association of variants with gene expression in the brain

The associations between AD risk allele carrier status and brain gene expression are shown in Additional file 1: Table S4. Figure 1 shows the significantly associated variants in 10 different brain regions. In total, 18 variants were detected to be associated with the altered expression of 15 nearby genes in specific brain regions. Especially in AD-related TCTX, HIPP, and FCTX regions, the minor alleles of rs11136000 in *CLU* and rs6701713 in *CR1* were associated with increased expressions of CLU and CR1. The minor alleles of rs2526378 in *BZRAP1-AS1* and rs12590654 in *SLC24A4/RIN3* were also associated with increased expression especially in TCTX/FCTX and FCTX, respectively, while the minor allele of rs3865444 in *CD33* was associated with decreased expression in TCTX (Additional file 1: Figure S2).

### Association of variants with brain amyloidosis

**Fig. 2:**
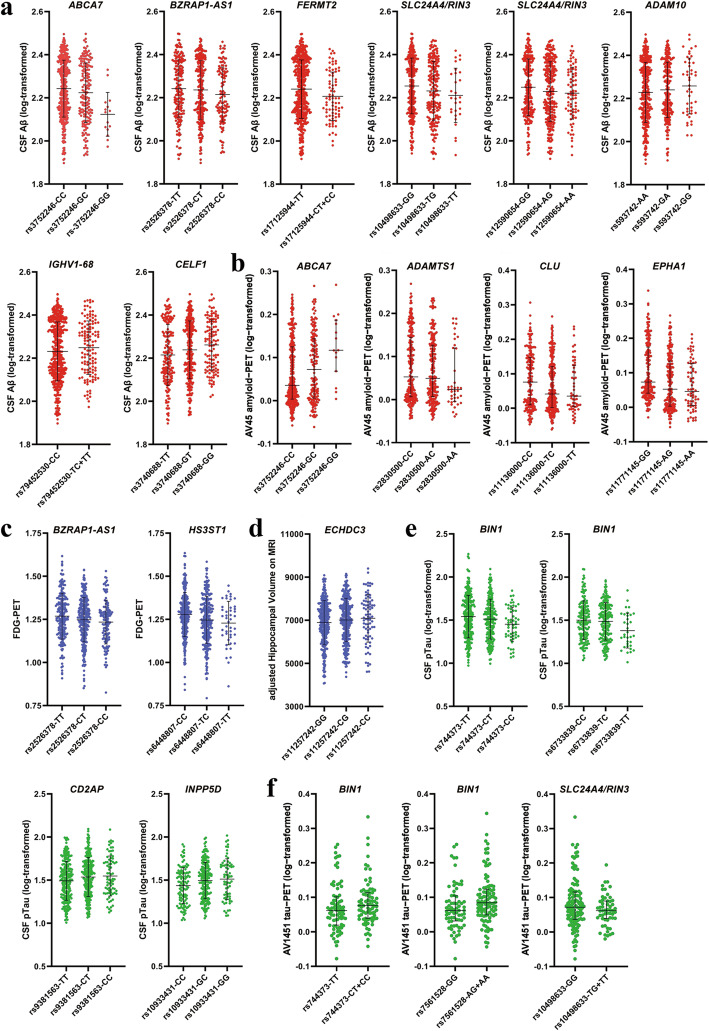
Association of the variants with brain amyloidosis, tauopathy, and neurodegeneration (FDG-PET levels or MRI hippocampal volumes) at baseline. We tested for significant associations of AD risk allele carrier status with brain amyloidosis, based on CSF Aβ_42_ or amyloid-PET data; the associations with brain neurodegeneration, based on FDG-PET or MRI HVa data; and the associations with brain tauopathy, based on CSF pTau or tau-PET data. **a** The minor allele carriers of *ABCA7* rs3752246, *BZRAP1-AS1* rs2526378, *FERMT2* rs17125944, *SLC24A4/RIN3* rs10498633, and *SLC24A4/RIN3* rs12590654 were significantly associated with decreased CSF Aβ_42_ levels, and *ADAM10* rs593742, *IGHV1-68* rs79452530, and *CELF1* rs3740688 associated with increased CSF Aβ_42_ levels. **b** The minor allele carriers of *ABCA7* rs3752246 were associated with increased amyloid-PET levels, and *ADAMTS1* rs2830500, *CLU* rs11136000, and *EPHA1* rs11771145 associated with decreased levels of amyloid-PET. **c** The minor allele carriers of *BZRAP1-AS1* rs2526378 and *HS3ST1* rs6448807 were associated with decreased FDG-PET levels. **d** The minor allele carriers of *ECHDC3* rs11257242 were associated with increased MRI HVa levels. **e** The minor allele carriers of *BIN1* rs744373 and *BIN1* rs6733839 had lower CSF pTau levels, and *CD2AP* rs9381563 and *INPP5D* rs10933431 had higher CSF pTau levels. **f** The minor allele carriers of *BIN1* rs744373 and *BIN1* rs7561528 had higher tau-PET levels, and *SLC24A4/RIN3* rs10498633 had lower tau-PET levels

**Fig. 3:**
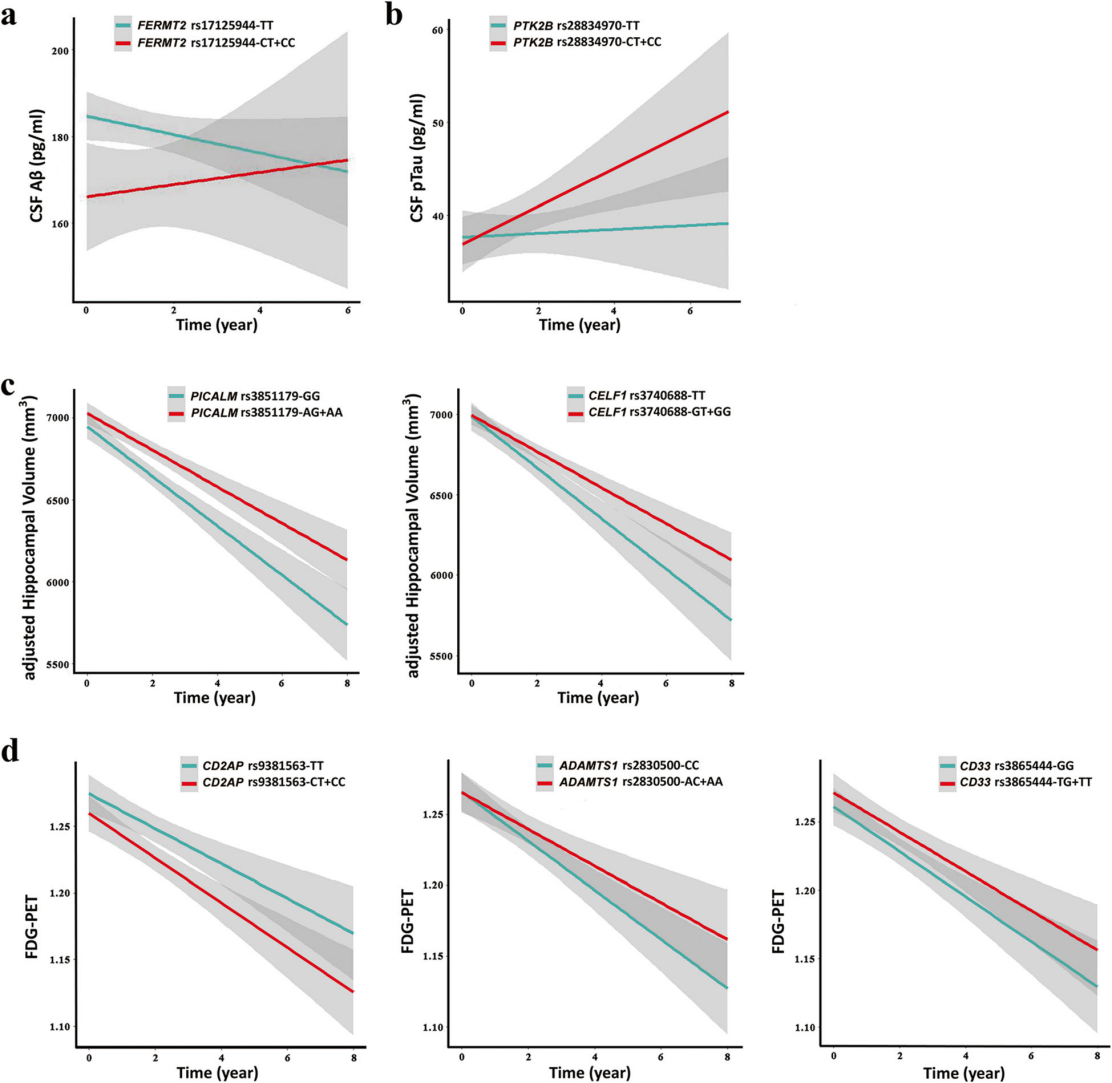
Longitudinal effects of the variants on the change rates of brain amyloidosis, tauopathy, and neurodegeneration. **a** The risk C allele carrier of *FERMT2* rs17125944 was associated with decreased CSF Aβ_42_ levels at baseline, although these changes over time were not obvious from the longitudinal analysis. **b** The longitudinal effect of *PTK2B* rs28834970 on the change rate of CSF pTau levels was significant. The minor allele carriers of *PTK2B* rs28834970 exhibited faster rise in CSF pTau levels. **c** Longitudinal effects of *CELF1* rs3740688 and *PICALM* rs3851179 on the change rate of MRI HVa levels were significant. The minor allele carriers of *CELF1* rs3740688 and *PICALM* rs3851179 exhibited slower decline in MRI HVa levels. **d** Longitudinal effects of *ADAMTS1* rs2830500, *CD2AP* rs9381563, and *CD33* rs3865444 on the change rate of FDG-PET levels were significant. The minor allele carriers of *CD2AP* rs9381563 exhibited faster decline in FDG-PET levels, and the minor allele carriers of *ADAMTS1* rs2830500 and *CD33* rs3865444 exhibited slower decline in FDG-PET levels. We analyzed the longitudinal effects of these variants on the change rates of pathology using a linear mixed effect model. The shaded regions refer to 95% confidence intervals

We tested for associations of AD risk allele carrier status with brain amyloidosis, based on CSF Aβ_42_ or amyloid-PET data at baseline and follow-up. Our results indicate that *ABCA7* rs3752246 was significantly associated with CSF Aβ_42_ levels (FDR-adjusted *P =* 0.008) and amyloid-PET levels (FDR-adjusted *P =* 0.001) at baseline (Fig. 2a, b), suggesting the strongest association with brain amyloid pathology. In addition, *FERMT2* rs17125944 was detected as associated with the altered CSF Aβ_42_ levels at baseline and follow-up. The risk C allele carrier of rs17125944 was associated with decreased CSF Aβ_42_ levels at baseline (FDR-adjusted *P =* 0.004), but these changes over time were not clear from the longitudinal analysis (Fig. 3a).

There were 9 other variants associated with brain amyloidosis. FDR-adjusted *P* values are shown in Additional file 1: Table S5. Among them, *ADAM10* rs593742, *BZRAP1-AS1* rs2526378, *CELF1* rs3740688, *IGHV1-68* rs79452530, *SLC24A4/RIN3* rs10498633, and *SLC24A4/RIN3* rs12590654 were associated with altered CSF Aβ_42_ levels at baseline (Fig. 2a), while *ADAMTS1* rs2830500, *CLU* rs11136000, and *EPHA1* rs11771145 were associated with altered amyloid-PET levels at baseline (Fig. 2b). Remarkably, there was no evidence for an effect of these variants on CSF Aβ_42_ levels or amyloid-PET levels from the longitudinal analysis.

### Association of variants with brain tauopathy

We examined the associations of AD risk allele carrier status with brain tauopathy, based on CSF pTau or tau-PET data at baseline and follow-up. Our results indicate that *BIN1* rs744373 was significantly associated with CSF pTau levels (FDR-adjusted *P =* 0.004) and tau-PET levels (FDR-adjusted *P =* 0.045) at baseline (Fig. 2e, f), and thus, there is a strong association with brain tau pathology. In addition, another two variants rs6733839 and rs7561528 in *BIN1* were detected to be associated with tau pathology. The minor allele carriers of rs744373 and rs6733839 in *BIN1* had lower CSF pTau levels at baseline (Fig. 2e), and rs744373 and rs7561528 in *BIN1* had higher tau-PET levels (Fig. 2f).

Furthermore, *CD2AP* rs9381563 and *INPP5D* rs10933431 were discovered to be associated with altered CSF pTau levels, and *SLC24A4/RIN3* rs10498633 was associated with altered tau-PET levels at baseline (Fig. 2e, f). Additional file 1: Table S5 lists the FDR-corrected *P* results for these associations. Based on the longitudinal follow-up data, although there was no association of *PTK2B* rs28834970 with brain tauopathy at baseline, the longitudinal effect of this variant on the change rate of CSF pTau levels was significant (FDR-adjusted *P =* 0.018). The minor allele carrier of *PTK2B* rs28834970 was associated with the rapid growth rate of CSF pTau levels (Fig. 3b; Additional file 1: Figure S3a).

### Association of variants with brain neurodegeneration

We analyzed the associations of AD risk allele carrier status with brain neurodegeneration, based on FDG-PET or MRI HVa data at baseline and follow-up. The newly discovered variants *BZRAP1-AS1* rs2526378 and *HS3ST1* rs6448807 were associated with altered FDG-PET levels (FDR-adjusted *P =* 0.046 and *P =* 0.007, respectively; Fig. 2c), and *ECHDC3* rs11257242 associated with altered MRI HVa levels (FDR-adjusted *P =* 0.045, Fig. 2d) at baseline. Based on the longitudinal follow-up data, the longitudinal effects of *CELF1* rs3740688 and *PICALM* rs3851179 on the change rate of MRI HVa levels were significant, and the effects of *ADAMTS1* rs2830500, *CD2AP* rs9381563, and *CD33* rs3865444 on the change rate of FDG-PET levels were also significant, although there was no association of these variants with brain neurodegeneration at baseline. Figure 3c, d shows these longitudinal associations (also in Additional file 1: Figure S3b,c), and Additional file 1: Table S5 lists the FDR-corrected *P* results.

**Fig. 4:**
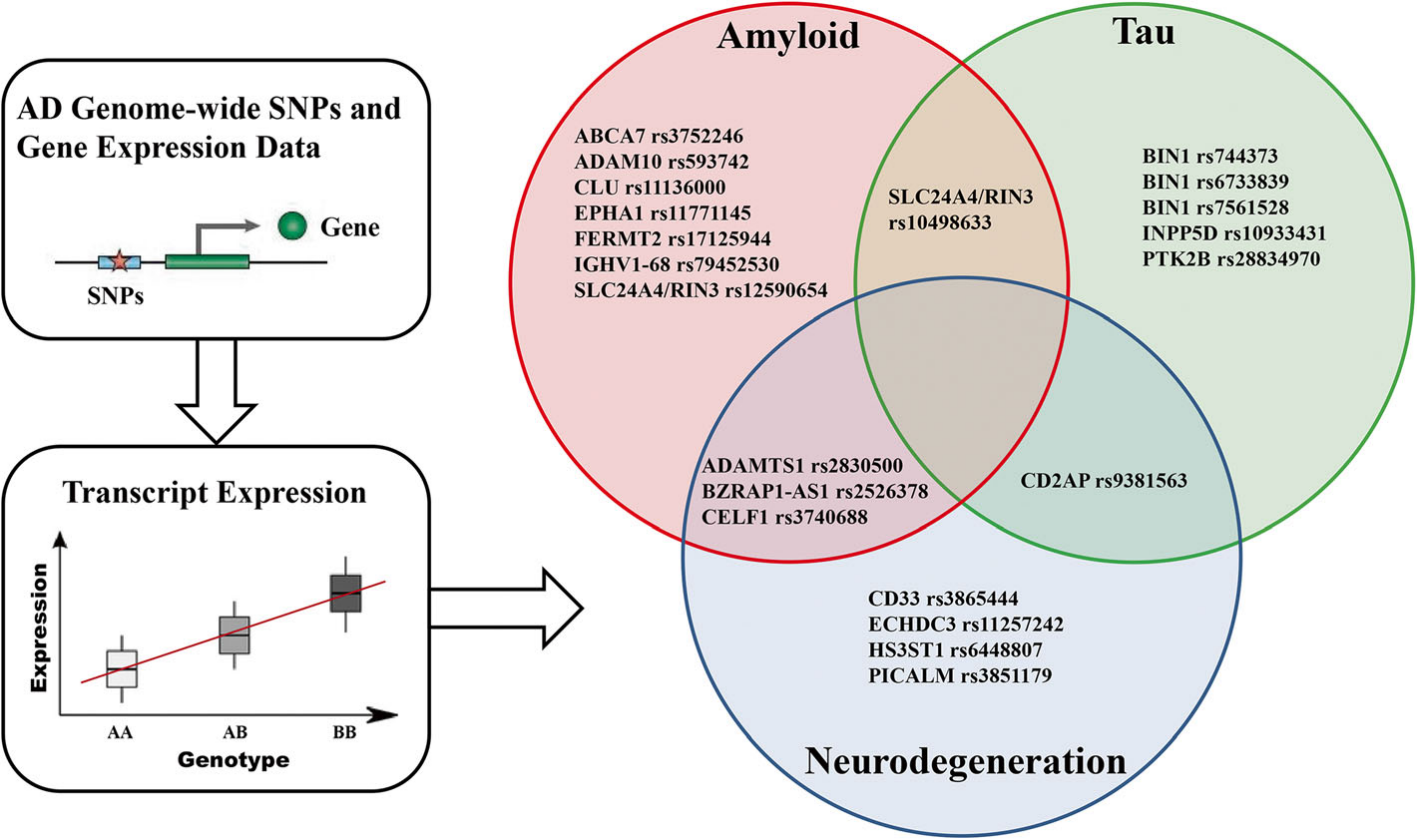
Role of variants in top 30 non-*APOE* AD risk genes in AD pathogenesis. Although most AD-associated genetic variants described to date are located in intronic or noncoding regions, these variants still could affect the nearby gene expression and exert protective or disease-inducing effects in AD-related amyloidosis, tauopathy, or neurodegeneration. Based on our analysis of all currently available data in ADNI, 11 variants were associated with brain amyloidosis, 7 variants associated with brain tauopathy, and 8 variants associated with brain neurodegeneration. Among them, variants in *ADAMTS1*, *BZRAP1-AS1*, and *CELF1* affect the mechanisms involved both in brain amyloidosis and neurodegeneration, *CD2AP* in brain tauopathy and neurodegeneration, and *SLC24A4/RIN3* in brain amyloidosis and tauopathy, implying these genes might contribute to AD risk via either common or distinct mechanisms

It is worth noting that, according to our current set of analyses, AD-associated genetic variants *ADAMTS1* rs2830500, *BZRAP1-AS1* rs2526378, and *CELF1* rs3740688 might affect the mechanisms involved both in brain amyloidosis and neurodegeneration, implying the commonalities or convergence in function. AD-associated genetic variants *CD2AP* rs9381563 might affect the mechanisms involved both in brain tauopathy and neurodegeneration, and *SLC24A4/RIN3* rs10498633 in brain amyloidosis and tauopathy (Fig. 4).

## Discussion

Although large GWAS has recently identified novel variants that are associated with altered AD risk, we have a relatively poor understanding of their functional impact. In this study, we comprehensively analyze possible functional effects of the variants in top 30 non-*APOE* AD risk genes, based on whether (1) these variants are associated with altered expression levels and (2) the variants are associated with brain amyloidosis, tauopathy, and neurodegeneration.

Firstly, 27 variants were detected as associated with the altered expression of 21 nearby genes in blood and brain regions. Among them, variants in *ABCA7*, *ADAM10*, *ADAMTS1*, *BZRAP1-AS1*, *CASS4*, *CD2AP*, *CD33*, *CELF1*, *CLU*, *HS3ST1*, and *PICALM* were associated with the altered gene expressions in different brain regions alone, and variants in *BIN1*, *ECHDC3*, *EPHA1*, *HLA-DRB1*, *NME8*, and *PTK2B* were associated with the altered gene expressions only in the blood, while only 6 variants in *CR1*, *MS4A6A*, *SLC24A4/RIN3*, and *ZCWPW1* affected these 4 nearby gene expressions both in the blood and in the brain, which perhaps become promising key biomarkers for AD diagnosis. Previous studies have shown that the overlap of genomic variants influencing transcript expression levels in both the human brain and blood is relatively low [24]. Of course, the smaller sample sizes from human brain tissues might be one possible reason for an underestimation of the true level. Notably, although 6 variants are associated with both brain and blood expression, the association was not always in the same direction. For example, *CR1* rs6701713 was associated with decreased expression in blood but increased expression in specific brain regions. The direction of association of the same variant in different brain regions was also different (Additional file 1: Figure S2). Therefore, genetic variants may require tissue-, cell-, region-, and disease-specific factors to exert their influences on gene expression. In any case, the variants associated with AD susceptibility are more likely to affect the expression levels in a tissue-specific and region-specific manner, and provide important regulating mechanisms of genetic variants in AD risk.

AD research has mainly focused in brain TCTX, HIPP, and FCTX regions. Consistent with previous studies [12, 25–27], our study showed that AD risk variants in *CR1*, *CD33*, *CLU*, and *SLC24A4/RIN3* affect their gene expression levels in the above key regions for brain regulatory effects. Remarkably, the novel variant rs2526378 in *BZRAP1-AS1* was found to influence its expression levels in TCTX/FCTX. Besides the changes in cortical regions, some genetic variants were associated with the altered expression in subcortical brain structures (THAL, PUTM) or WHMT (Fig. 1). Because the degenerative processes in these regions might contribute to cognitive decline and are mechanistically important in AD [28–30], more attention should be paid to these associations in future research, which might become novel potential treatment targets. In general, evaluating the potential associations using large-scale expression GWAS datasets for multiple brain regions and peripheral blood in the same individuals would provide more valuable information.

Based on the baseline and longitudinal follow-up data, we were able to confirm the previously reported associations of genetic variants in *ABCA7*, *CELF1*, *CLU*, *EPHA1*, and *FREMT2* with brain amyloidosis as previously described [16, 20, 31]. The novel genome-wide variants in *ADAM10* (most important α-secretase in the process of amyloid-β protein precursor (APP) cleavage) [32, 33] and *ADAMTS1* (within 665 kb of *APP* on chromosome 21) [34] were also found to affect brain amyloidosis. Additionally and to the best of our knowledge, we are the first to report the associations for novel variants in *IGHV1-68*, *BZRAP1-AS1*, and *SLC24A4/RIN3* with brain amyloidosis.

Previous studies have shown that *BIN1* rs744373 was associated with altered tau-PET and CSF pTau levels [18, 35]. Our study supports the suggestion that other variants in *BIN1* are also significantly associated with tau biomarkers. Similarly, we confirm the previously reported association of *INPP5D*, *CD2AP*, and *PTK2B* with brain tauopathy [19, 20, 36, 37]. Conversely, the relationship between the variant in *SLC24A4/RIN3* and tau pathology is a new discovery. *SLC24A4* CpG methylation sites were found associated with Aβ burden and tau pathology previously [38] and *SLC24A4* also appeared to take part in lipid metabolism [39] and brain glucose metabolism [17].

As most of the earlier studies were cross-sectional analyses, a stage-specific association might occur for genetic variants that influence the course of neurodegeneration [17]. Using longitudinal follow-up data in our analysis, it is possible to identify the effects of variants in *CD2AP*, *CD33*, *CELF1*, and *PICALM* on the changes in brain metabolism or atrophy over time, supporting the reported associations of these genes with brain neurodegeneration [17, 40–42]. However, the associations of variants in *ADAMTS1*, *ECHDC3*, *BZRAP1-AS1*, and *HS3ST1* with neurodegeneration biomarkers at baseline are novel findings of this research. Aligned with this, *HS3ST1* was reported as significantly associated with working memory in probable-MCI patients [43].

According to our results, variants in *ADAMTS1*, *BZRAP1-AS1*, and *CELF1* affect the mechanisms involved both in brain amyloidosis and neurodegeneration, *CD2AP* in brain tauopathy and neurodegeneration, and *SLC24A4/RIN3* in brain amyloidosis and tauopathy (Fig. 4), implying these genes might contribute to AD risk via either common or distinct mechanisms. Previous studies have shown that *ADAMTS1*, within 665 kb of *APP* on chromosome 21, has elevated expression in Down’s syndrome and LOAD brain [34, 44] and is a potential neuroprotective gene or neuroinflammatory gene important to microglial response [45]. *BZRAP1* (also known as TSPOAP1) is a subunit of the benzodiazepine receptor complex in mitochondria and a marker of neuroinflammation. Previous studies have demonstrated that the TSPO ligand can reverse Aβ accumulation and behavioral impairment in transgenic mice [46]. Moreover, the *CELF1* variant has been shown to affect cognition and CSF Aβ_42_ levels by modifying expression [31, 47]. And the fly homolog of *CELF1*, *aret*, also shows mediation of tau toxicity [48]. Much more research on the roles of these genes and how they relate to each other is very important.

It is worth noting that, in our study, we place CSF and PET imaging biomarkers all into analysis; that is because the fundamental difference between the two should be recognized. CSF biomarkers are measures of the concentrations of proteins in CSF from the lumbar sac that reflect the rates of both production (protein expression or release/secretion from neurons or other brain cells) and clearance (degradation or removal) at a given point in time. While imaging measures, on the other hand, represent the magnitude of the neuropathologic load or damage accumulated over time. Low CSF Aβ_42_ is therefore best considered a biomarker of a pathologic state that is associated with amyloid plaque formation and not a measure of amyloid plaque load as amyloid-PET is. Similarly, CSF pTau is best considered a biomarker of a pathologic state that is associated with PHF tau formation and not a measure of pathologic tau deposits as tau-PET is [15]. In addition, FDG-PET was labeled as neurodegeneration biomarkers in our current study, because our selection and classification of biomarkers is based on the 2018 NIA-AA research framework. However, a growing number of studies, including the recent article published by our team [49], suggest FDG-PET as an independent biomarker for Alzheimer’s biological diagnosis, because FDG hypometabolism is a summation of multiple biological processes, not just neuronal hypometabolism and neurodegeneration. We sincerely hope more practitioners and academia to deep study in this field to further improve the understanding of AD biomarkers.

Generally, the ultimate goal of understanding the genetic architecture of AD is to enhance the understanding of disease mechanisms. Based on the above A/T/N classification system, our identification of many risk genes suggests their shared function in brain amyloidosis, tauopathy, or neurodegeneration, which might provide interesting targets for future functional follow-up and biological interpretation.

## Limitation

Our current study has limitations. Firstly, the subjects with tau-PET data, especially the longitudinal tau-PET data, are limited. Secondly, the follow-up CSF data are limited because of the invasiveness of the procedure to acquire samples. With the rapid development and wide application of PET technology, future studies will validate our findings in a large, independent, longitudinal cohort with a greater number of individuals and time points and with a longer follow-up time to provide more statistically powerful results.

## Conclusions

In summary, our current study provides new insights into the variants in the top 30 non-*APOE* AD risk genes associated with transcript expression levels and involved in the pathological processes of brain amyloidosis, tauopathy, and neurodegeneration. This evidence increases the possibility that genetic variants might play functional roles and suggest potential mechanisms in AD pathogenesis. Further studies are needed to fully understand their roles in AD process, and our research opens doors to the investigation of novel targets for AD treatment.

## Supplementary Information

**Additional file 1.**

## Data Availability

The datasets used and/or analyzed during the current study are available from the corresponding author on reasonable request.

## Abbreviations

AD: Alzheimer’s disease
ADNI: Alzheimer’s Disease Neuroimaging Initiative
CSF: Cerebrospinal fluid
FDG: Fluorodeoxyglucose
GWASs: Genome-wide association studies
HV: Hippocampal volume
LOAD: Late-onset Alzheimer’s disease
MCI: Mild cognitive impairment
NC: Normal cognition
NIA-AA: National Institute on Aging and Alzheimer’s Association
PET: Positron-emission tomography
pTau: Phosphorylated tau
tTau: Total tau
UKBEC: UK Brain Expression Consortium
